# Prostate adenocarcinoma in HIV-associated type 6 pityriasis rubra pilaris: A case report

**DOI:** 10.1016/j.jdcr.2026.05.018

**Published:** 2026-05-13

**Authors:** Andre Skipper, Nirav Saini, Charlene Hunter, Lynne H. Robertson

**Affiliations:** a Cumming School of Medicine, University of Calgary, Calgary, Alberta, Canada; b Department of Pathology, University of Calgary, Calgary, Alberta, Canada; c Division of Dermatology, Department of Medicine, Cumming School of Medicine, University of Calgary, Calgary, Alberta, Canada

**Keywords:** acquired immunodeficiency syndrome, antiretroviral therapy, HIV infection, immunocompromised host, pityriasis rubra pilaris, prostate adenocarcinoma, skin diseases

## Introduction

Human immunodeficiency virus (HIV)-associated pityriasis rubra pilaris (PRP), also known as type 6 PRP, is a rare diagnosis with an incidence of less than 1 in 5000 patients.^1^ PRP has emerged in case reports as a potential indication of a neoplastic disorder with complete to near-complete resolution following treatment.^2^

Although typically refractory to mainstay therapies, including methotrexate and acitretin, recent advances suggest that interleukin-17a monoclonal antibodies, tumour necrosis factor-alpha inhibitors, and combination antiretroviral therapies may provide clinical benefit.^3^ We report a case of Type 6 PRP in a stable HIV-positive male patient on antiretroviral therapy who was subsequently diagnosed with high grade prostate adenocarcinoma.

## Case report

A 69-year-old male with longstanding, well-controlled HIV (diagnosed in 2002; CD4 nadir 230 cells/mm^3^) presented with a 5-month history of a generalized pruritic dermatosis that started on the head and neck and progressed cephalocaudally. This was unresponsive to 7 to 10-day courses of terbinafine, doxycycline, and itraconazole as well as topical betamethasone valerate 0.1% cream which had been trialed prior to referral. Comorbidities included chronic obstructive pulmonary disease, dyslipidemia, and mild coronary artery disease. His medications at presentation were Dovato (dolutegravir/lamivudine), Anoro-Ellipta (umeclidinium/vilanterol), salbutamol, and acetylsalicylic acid 81 mg.

**Fig 1:**
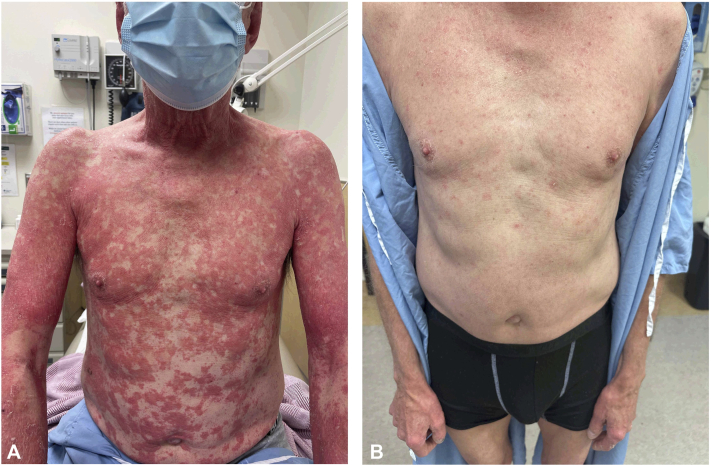
Pityriasis rubra pilaris presenting on the ventral trunk of a 69-year-old male with a history of long-standing, stable HIV with a high-grade adenocarcinoma of the prostate. Significant improvement was noted after 3 y of treatment with acitretin, ixekizumab and management of an underlying prostate adenocarcinoma. **(A)**. Patient chest prior to treatment and **(B)**. Patient’s chest demonstrating cutaneous improvement 3 years following treatment. *HIV*, Human immunodeficiency virus.

**Fig 2:**
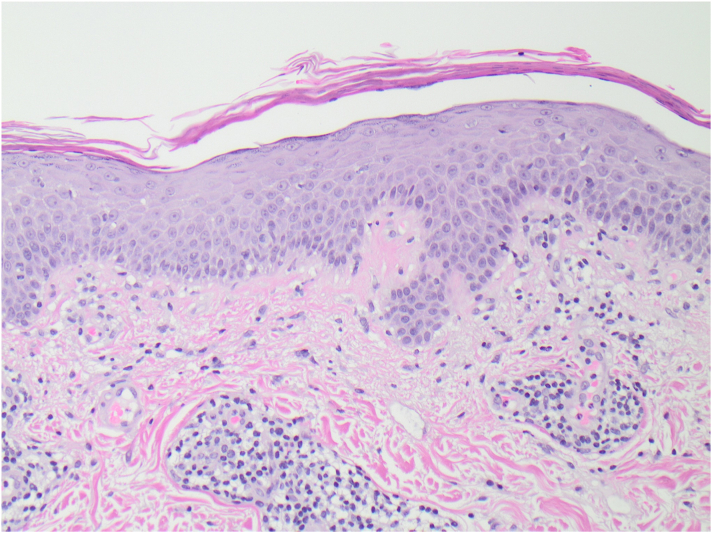
Hyperkeratosis alternating with parakeratosis, mild spongiosis, and a superficial perivascular lymphocytic infiltrate (hematoxylin and eosin; 20×).

On initial evaluation the patient had hemodynamically stable erythroderma, ectropion, and palmoplantar keratoderma. The dermatosis manifested as scaly orange-erythematous papules and plaques, interspersed with small islands of normal skin (Fig 1, *A*). Histologic sections showed parakeratosis alternating with orthokeratosis overlying mild epidermal acanthosis and spongiosis (Fig 2). There was slight follicular plugging. The granular layer was intact. Within the dermis, there was a superficial perivascular lymphocytic infiltrate. Eosinophils were not a significant finding. There were slightly dilated vessels at the tips of dermal papillae but significant thinning of the suprapapillary plates was not seen. Further investigations including a CBC, liver function studies, creatinine, electrolyte panel, TSH, and SPEP were normal. Tests for syphilis and tuberculosis (QuantiFERON gold) were negative, however, serology for toxoplasmosis was positive. Hepatitis screening demonstrated hepatitis B core antibody positivity and negative Hepatitis B surface antigen. Absolute CD4+ and CD8+ T-lymphocyte counts were normal. The HIV viral load was undetectable. The collective clinical and histopathologic findings were consistent with a diagnosis of HIV-associated PRP.

Initial treatment included ixekizumab and acitretin. In this case, interleukin-17A inhibition was sought with ixekizumab in light of increasing evidence suggesting the contribution of Th17/interleukin-17 signaling to keratinocyte-driven inflammation. The ixekizumab was held after 2 doses and restarted 2 months later following confirmation there was minimal risk of hepatitis B and toxoplasmosis reactivation through an infectious disease consultation. While the patient's PRP improved over the first 2 months, relapse was noted prior to the reinitiation of the biologic. After 18 m, he achieved approximately 85% clearance (Fig 1, *B*), with residual mild keratoderma; and this improvement persisted at 3 years, with 90% clearance noted. Despite the absence of constitutional B symptoms, the prostate specific antigen was elevated (16.2 μg/L) and a prostate biopsy revealed high grade, Gleason grade group 5/5, Gleason score 9/10 (4 + 5), adenocarcinoma. A computed tomography chest, abdomen and pelvis showed no evidence of metastatic disease. Ixekizumab and acitretin were continued while receiving brachytherapy and external beam radiation therapy for the prostate cancer following which time there was almost 100% clearance of the PRP (Fig 1, *B*).

## Discussion

The first documented case of Type 6 PRP was reported in 1991 by Dr Blauvelt et al.^4^ It is typically characterized by a cephalocaudal spread of erythematous-to-orange scaly plaques, with islands of well-defined sparing in the setting of HIV infection. Palmoplantar keratoderma, as well as nail changes, may be present. Additional clinical features described with Type 6 PRP, but not seen in our patient, include acne conglobata, hidradenitis suppurativa, and lichen spinulosus. The etiology is unclear; however, immune dysregulation caused by HIV is thought to play a role.^5^

A review of the literature revealed that all previously reported cases of Type 6 PRP occurred within 6 years of HIV diagnosis, with most presenting within 3 to 4 years (Table I). Interestingly, only 4 female cases were identified and twelve of nineteen cases reported hidradenitis suppurativa, acne conglobata, and/or lichen spinulosus as comorbidities (Table I). Our case represents the longest interval at 20 y. This extended latency may expand the window during which clinicians consider Type 6 PRP in the differential, including amongst patients with well-controlled HIV. No clear correlation exists between CD4 count and PRP onset, as cases range from severe immunosuppression (CD4 <50 cells/μL) to near-normal immune function (CD4 > 500 cells/μL). Additionally, one case demonstrated spontaneous PRP resolution despite poor treatment adherence, raising the possibility that some Type 6 PRP cases may be self-limiting.^6^

**Table I:** Review of type 6 PRP cases – adapted from Dipanker et al

Patient	Authors	Age/sex	Sexual preference	HIV diagnosis before/after PRP	Time between HIV and PRP diagnosis	CD4 T-cell count (cells/mm^3^)	Viral load (copies/mL)	Cutaneous lesions	HIV treatment	PRP therapy	Subsequent status
1	Blauvelt et al	28 M	Bisexual	After	NR	368	NR	Plaques PPK	AZT	UVB Etretinate	No improvement on AZT Slight improvement on Etretinate
2	Blauvelt et al	28 M	Homosexual	Before	2 mo	311	NR	AC, LS, nail involvement, plaques, palmoplantar scaling	AZT TMP-SFX	Isotretinoin Etretinate PUVA UVB	Side effects NR Slight Improvement
3	Auffret et al	28 F	NR	Before	2 y	714	NR	AC, LS, PPK, HS, plaques, nail involvement	AZT	Isotretinoin Etretinate	Minimal improvement Discontinued due to SE's Died of pulmonary infection and shock
4	Resnick et al (not labeled as PRP)	45 M	NR	After	NR	510	NR	AC, LS	AZT Minocycline	Prednisolone Isotretinoin	Limited Improvement Exacerbation followed by relapse 50% improvement with residual scarring
5	Menni et al	4 M	NR	Before	4 y	1140	NR	PPK, keratotic scaly papules	AZT		Cleared with residual hypopigmentation
6	Martin et al	29 M	Homosexual	Before	3 mo prior	>500	NR	AC, LS, acuminate follicular papules	Minocycline	Isotretinoin Etretinate UVB	No improvement No improvement Complete clearing
7	Martin et al	40 M	Bisexual	Before	NR (Reported as several years prior)	NR	NR	AC, LS, PPK, follicular hyperkeratotic plaques	NR	Isotretinoin Methotrexate Etretinate	Some improvement Stopped due to complications Considerable improvement
8	Martin et al	27 M	Homosexual	Before	6 mo	699	NR	PPK, plaques	AZT	Corticosteroids Etretinate	No improvement Partial clearing 95% clearing
9	Perrin et al	31 M	NR	NR	NR	8	NR	LS, papular eruption, slight PPK	NR	NR	No improvement
10	Sanchez- Regaña et al.	35 M	NR	After	NR	86	NR	PPK, keratotic acuminate follicular papules, plaques	AZT	Corticosteroids Etretinate	Complete clearance for 1 year Returned and was treated with etretinate with slight improvement
11	Miralles et al	32 M	Homosexual	After	NR	597	NR	AC, PPK, nail changes, follicular keratotic papules, plaques	AZT Clindamycin	Isotretinoin Prednisone AZT Etretinate	No improvement
12	Bonomo et al	36 M	NR	Before	6 mo	200	NR	PPK, nail involvement, psoriaform erythroderma	AZT Stavudine	Methotrexate Isotretinoin UVA Etretinate	No improvement
13	Gonzalez-Lopez et al	34 F	NR	Before	3 y	309	9582	AC, follicular papules with plugs and hyperkeratotic spicules	AZT Lamivudine Saquinavir	NR	Complete clearing
14	Basdale et al	24 M	Homosexual	Before	15 mo	600	>750,000	AC, PPK, HS, plaques, nail involvement	Indinavir Stavudine 3 TC	Isotretinoin	Complete clearing with residual scarring and non-adherence to treatment
15	Dipankar et al	45 M	NR	After	NR	NR	NR	AC, LS, plaques, nail involvement	NR	Isotretinoin	Lost to follow-up
16	Lerebours-Nadal et al.	23 F	NR	Before	3 y	500	NR	HS, LS, scaly rash with islands of sparing, nail involvement	Stavudine 3 TC Efavirenz AZT TMP-SFX	NR	Complete clearing with severe residual scarring
17	Nair et al	30 M	NR	After	NR	NR	NR	Plaques, nail involvement	Unspecified ART	Methotrexate	NR Complete clearance
18	Williams et al	24 F	NR	Before	6 y	192	NR	AC, PPK, Hyperkeratotic follicles and plaques	Acitretin AZT 3 TC Nevirapine	NR	Complete clearance
19	Kranyak et al	53 M	NR	NR	4 y	407 (5 mo prior to PRP presentation)	Undetectable (<20)	Erythrodermic rash, follicular papules and plaques	NR	Ixekizumab	Complete clearance

*3 TC*, Lamivudine; *AC*, acne conglobata; *AZT*, zidovudine; *F*, female; *HS*, hidradenitis suppurativa; *LS*, lichen spinulosus; *M*, male; *NR*, not reported; *NVP*, nevirapine; *PPK*, palmoplantar keratoderma; *PRP*, pityriasis rubra pilaris.

While PRP is not traditionally considered a paraneoplastic syndrome, its association with malignancy has been documented in isolated cases. Supplementary Table I (available via Mendeley at https://doi.org/10.17632/g5wk3g8bd.1) outlines literature showing malignancies that have been associated with PRP. Out of 17 cases of malignancy associated with PRP, 10 resolved during or soon after treatment of the malignancy.^5^ The mechanism linking PRP to malignancy is unclear. Our case underscores the possibility of an underlying paraneoplastic mechanism contributing to disease persistence and treatment resistance.

In summary, we present a case of type 6 PRP in a patient with longstanding, well-controlled HIV, who was found to have an underlying genitourinary malignancy. The extended latency of the presentation challenges the previously observed pattern of PRP onset predominantly within 5 years of HIV diagnosis and suggests type 6 PRP may occur in stable, long-term managed patients. Additionally, this case supports the potential effectiveness of ixekizumab in type 6 PRP, with an initial therapeutic response followed by symptom recurrence upon temporary discontinuation. To our knowledge, this case is unique in that there are no prior reports of PRP occurring in the setting of both HIV and malignancy. It highlights the importance of screening for both HIV and malignancy in patients with PRP that is refractory to therapy.

## Conflicts of interest

None disclosed.
